# Clinical study of a survivin long peptide vaccine (SurVaxM) in patients with recurrent malignant glioma

**DOI:** 10.1007/s00262-016-1890-x

**Published:** 2016-08-30

**Authors:** Robert A. Fenstermaker, Michael J. Ciesielski, Jingxin Qiu, Nuo Yang, Cheryl L. Frank, Kelvin P. Lee, Laszlo R. Mechtler, Ahmed Belal, Manmeet S. Ahluwalia, Alan D. Hutson

**Affiliations:** 1 Department of Neurosurgery, Roswell Park Cancer Institute, Elm and Carlton Streets, Buffalo, NY 14263 USA; 2 Department of Pathology, Roswell Park Cancer Institute, Elm and Carlton Streets, Buffalo, NY 14263 USA; 3 Department of Neuro-Oncology, Roswell Park Cancer Institute, Elm and Carlton Streets, Buffalo, NY 14263 USA; 4 Department of Diagnostic Imaging, Roswell Park Cancer Institute, Elm and Carlton Streets, Buffalo, NY 14263 USA; 5 Department of Immunology, Roswell Park Cancer Institute, Elm and Carlton Streets, Buffalo, NY 14263 USA; 6 Department of Biostatistics, Roswell Park Cancer Institute, Elm and Carlton Streets, Buffalo, NY 14263 USA; 7 Center for Immunotherapy, Roswell Park Cancer Institute, Elm and Carlton Streets, Buffalo, NY 14263 USA; 8 Burkhardt Brain Tumor and Neuro-Oncology Center, Cleveland Clinic Foundation, Cleveland, OH 44195 USA

**Keywords:** Apoptosis, Glioma, Immunotherapy, Peptide, Survivin, Vaccine

## Abstract

Survivin is an anti-apoptotic protein that is highly expressed in many cancers, including malignant gliomas. Preclinical studies established that the conjugated survivin peptide mimic SurVaxM (SVN53-67/M57-KLH) could stimulate an anti-tumor immune response against murine glioma in vivo, as well as human glioma cells ex vivo. The current clinical study was conducted to test safety, immunogenicity and clinical effects of the vaccine. Recurrent malignant glioma patients whose tumors were survivin-positive, and who had either HLA-A*02 or HLA-A*03 MHC class I allele-positivity, were given subcutaneous injections of SurVaxM (500 μg) in Montanide ISA 51 with sargramostim (100 μg) at 2-week intervals. SurVaxM was well tolerated with mostly grade one adverse events (AE) and no serious adverse events (SAE) attributable to the study drug. Six patients experienced local injection site reactions; three patients reported fatigue (grades 1 and 2), and 2 patients experienced myalgia (grade 1). Six of eight immunologically evaluable patients developed both cellular and humoral immune responses to vaccine. The vaccine also stimulated HLA-A*02, HLA-A*03 and HLA-A*24 restricted T cell responses. Three patients maintained a partial clinical response or stable disease for more than 6 months. Median progression-free survival was 17.6 weeks, and median overall survival was 86.6 weeks from study entry with seven of nine patients surviving more than 12 months.

## Introduction

Survivin (BIRC5) is a member of the inhibitor of apoptosis protein (IAP) family [1, 2]. Survivin expression in tumors is associated with a high rate of disease recurrence and resistance to chemotherapy, and it confers a significant survival advantage to tumor cells [3]. Its presence in gliomas and other tumors is reportedly associated with a poor prognosis [4]. It is expressed in all four subtypes of glioblastoma defined by The Cancer Genome Atlas (TCGA) project, including: classical, mesenchymal, neural and proneural variants [5].

Although expressed during fetal development [2], survivin is infrequently detected in the normal tissues of adult organisms [6]. Thus, the molecule represents a potential target for cancer immunotherapy. One immunohistochemical study of gliomas demonstrated that 29 of 29 glioma specimens (WHO grades II-IV), but not normal brain tissue, contain survivin-positive cells [7]. The mean percentage of cells detectable by immunohistochemical methods in each specimen was 70.0 % in grade II (low grade) gliomas, 81.3 % in grade III (anaplastic) gliomas and 85.0 % in grade IV gliomas (glioblastoma). Survivin is also present in association with plasma-derived exosomes of glioma patients in which its action remains to be defined [8].

As a largely intracellular protein, survivin is degraded by the proteasome and resulting epitopes are presented on the surface of tumor cells by MHC class I molecules. Accordingly, survivin-specific cytotoxic T lymphocytes (CTL) have been identified in some cancer patients [9, 10]. In addition to T cell-mediated immunity, many cancer patients develop humoral immune responses to survivin with anti-survivin antibodies detectable in serum [10]. Therefore, survivin is potentially immunogenic and such responses as these could provide the basis for induction of therapeutic anti-tumor immunity in cancer patients in whom the immune system is already primed to recognize survivin.

SurVaxM (SVN53-67/M57-KLH) contains a synthetic long peptide mimic that spans amino acids 53 through 67 of the human survivin protein sequence [11]. The amino acid alteration in this peptide (M57) leads to enhanced binding of the core survivin epitope to HLA-A*0201 molecules [11]. The peptide is conjugated to keyhole limpet hemocyanin (KLH), which acts as a vaccine adjuvant. The long peptide also contains multiple MHC class I epitopes that are presentable by other HLA molecules. In addition, the long peptide is able to stimulate specific CD4^+^ cytokine support [11, 12]. The presence of MHC class II-restricted CD4^+^ T cells that are specific for tumor-associated antigens has been recognized as an important element for providing helper factors essential for eliciting and sustaining cytotoxic CD8^+^ responses against tumors [13, 14].

## Patients and methods

### Study overview

This clinical study (clinicaltrials.gov identifier NCT01250470) was conducted in patients with HLA-A*02 and HLA-A*03 haplotypes who had histologically confirmed survivin-positive malignant gliomas that had recurred or progressed following standard therapy, including surgery, fractionated radiation therapy and chemotherapy with temozolomide. It was designed to test the toxicity (primary outcome) and immunogenicity (secondary outcome) of SurVaxM in emulsion with Montanide ISA 51 and given subcutaneously with sargramostim (GM-CSF) as a dendritic cell attractant and maturant. This was a nonrandomized, single-institution, clinical trial designed to assess a fixed dose vaccine regimen. Based on pre-clinical toxicity studies, a dose level of 500 μg SurVaxM was selected for testing. A regimen of four prime-boost doses at this level was given without dose escalation. In order to test the response to extended dosing, patients that survived 6 months without disease progression or regimen-limiting toxicity were eligible to receive additional booster doses of vaccine. The use of SVN53-67/M57-KLH in this study is registered with the USFDA under IND #14674 held by Roswell Park Cancer Institute (RPCI). The composition of SVN53-67/M57-KLH (SurVaxM) has been previously described [11]. All investigations were performed under a clinical protocol approved by the institutional review board at RPCI and in accordance with an assurance filed with and approved by the US Department of Health and Human Services. Informed consent was obtained from each subject prior to treatment.

### Patient eligibility

Inclusion criteria were: age ≥18 years, histologic proof of recurrent or progressive glioblastoma, anaplastic astrocytoma, anaplastic oligodendroglioma or anaplastic mixed glioma following failure of standard therapy, Karnofsky performance status (KPS) ≥70, HLA-A*02 or HLA-A*03 haplotype, survivin expression by tumor cells documented by immunohistochemistry, no systemic infection or ongoing antibiotic therapy, white blood cell count ≥3000/mm^3^, platelets ≥100,000/mm^3^, hemoglobin ≥10.0 g/dL, AST(SGOT)/ALT(SGPT) <2.5 × ULN, total bilirubin ≤2.0 mg/dL and serum creatinine ≤1.5× ULN. Patients with potential for child-bearing were required to agree to the use of acceptable contraceptive methods during treatment and for 3 months after receiving the last dose of vaccine, patients who had had recent cranial surgery were eligible for inclusion, but the vaccine could not be administered prior to the 14th post-operative day.

### Treatment plan

Nine patients were treated with SurVaxM (500 μg) in emulsion with Montanide ISA 51 with sargramostim (100 μg) every 2 weeks for a total of four doses per patient (prime-boost phase). All patients in each group were followed for at least 6 weeks before the next group of three patients could begin treatment to limit the total number of individuals exposed to possible toxicity. The dose of vaccine was not escalated in an individual patient or between cohorts. Patients were followed clinically every 2 weeks for the first 8 weeks and then monthly until tumor progression or death. Patients were assessed for regimen-limiting toxicity (RLT) using CTCAEv4.0 criteria (see definition of RLT below). Clinical responses were ascertained by neurologic exams on schedule and from serial MRI brain scans. Patients who survived 6 months without tumor progression, RLT or serious adverse event (SAE) were eligible to receive additional doses of the vaccine every 3 months (booster phase).

### Evaluation during study

The primary goal of this study was to assess the safety, tolerability and toxicity of the survivin peptide mimic vaccine regimen. A secondary goal was to measure immune responses to the vaccine, including anti-survivin antibody production, survivin-specific multimer immunoreactivity and IFNγ mRNA production following vaccination. Tertiary goals were to measure tumor progression after vaccination, identify any apparent treatment responses and measure survival. Safety was assessed by physical and neurological examinations and laboratory studies.

MRI brain scans were performed serially, and established criteria were used to determine radiographic response for those with measureable disease. Response categories included: (1) complete response (CR), disappearance of all enhancing tumor on consecutive MRI scans at least 1 month apart, off steroids, and neurologically stable or improved; (2) partial response (PR), 50 % reduction in size of enhancing tumor on consecutive MRI scans at least 1 month apart, steroids stable or reduced, and neurologically stable or improved; (3) progressive disease (PD), >25 % increase in size of enhancing tumor or any new tumor on MRI scans, or neurologically worse, and steroids stable or increased; and (4) stable disease (SD), all other situations. The measure of tumor size is the largest cross-sectional diameter multiplied by the largest diameter perpendicular to it. Progression-free survival (PFS) is defined as the duration of time from study entry to progression on MRI or death.

#### Clinical evaluability

Patients who received at least one dose of vaccine were considered evaluable for the purpose of determining toxicity. Patients who received ≥2 doses of vaccine were deemed evaluable for immunological response; however, those who did not receive ≥2 doses were not replaced (patient #4). Patients who received ≥3 doses of vaccine and had an MRI scan 8 weeks following initiation of therapy were considered evaluable for clinical response. Patients who were unevaluable for clinical response were not replaced (patient #4).

#### Safety

NCI common terminology criteria for adverse events (CTCAEv4.0) were used to evaluate toxicity. Toxicity was considered to be an adverse event possibly, probably or definitely related to treatment. The maximum grade of toxicity for each category of interest was recorded for each patient, and the summary results were tabulated by category and grade. Regimen-limiting toxicities (RLT) were defined to include: (1) any grade 3 or greater toxicity possibly, probably or definitely related to the vaccine, (2) any grade 2 or greater autoimmune disorder, (3) any grade 2 or greater allergic reaction and (4) any peptide vaccine dosing delay >2 weeks.

#### Survival

All patients were followed to ascertain both progression-free survival (PFS) and median overall survival (OS).

### Immunohistochemical studies

Immunohistochemical detection of survivin was performed using a Dako Omnis autostainer (Dako North America, Inc. Carpinteria, CA) with rabbit monoclonal survivin antibody clone EP119 (Bio SB, Santa Barbara, CA). Additional antibodies included: CD4 (4B12, Dako); CD8 (C8/144B, Dako); CD20 (L26, Dako); PD-L1/CD274 (SP142, Spring Bioscience). Stained specimens were viewed by the neuropathologist, co-investigator (JQ), and survivin expression in the nucleus and cytoplasm was determined to be present or absent.

### Immunologic assessments

#### Serum antibody measurements

Patient serum was collected and stored at −80 °C. Serial dilutions of clarified serum were applied to unconjugated survivin peptide, free KLH and random peptide (20 µg/ml, 1 µg/well) on pre-coated ELISA plates (Flat Bottom, Nunc) in triplicate. Samples were incubated at 4 °C overnight and washed (PBS, 1 % BSA). HRP-conjugated anti-human IgG detection antibody (Bio-Rad) was added for 1 h at 25 °C. Plates were washed four times, and TMB colorimetric solution (Biolegend) was added at room temperature and developed for 15 min and read on a Bio-Rad automated plate reader at 450 nm.

#### PBMC isolation

Peripheral blood mononuclear cells were isolated within 3 h from blood samples using Ficoll separation technique. Heparinized blood was diluted (1:1) with Hank’s balanced salt solution (HBSS) (Mediatech, Cat#: 21-023-CV). Blood/HBSS diluent was layered on top of lymphocyte separation medium (LSM; Mediatech, Cat#: 25-072-CV) in 50-ml conical tubes. Tubes were centrifuged at 400×*g* for 30 min. PBMC layer was centrifuged at 600×*g* for 10 min. Cell pellets were re-suspended in 5 ml of CTL wash solution (Cat#: CTLW010)/RPMI (Mediatech, Cat#: 10-040-CV) solution. The pooled sample was centrifuged at 500×*g* for 5 min, and the pellet was re-suspended in freezing media (10 % DMSO/90 % fetal bovine serum) and transferred to liquid nitrogen storage.

#### Multimer analysis

Custom-designed multimers included the following: MHC Dextramer-PE (Immudex, Copenhagen, Denmark) to A*0201:QMFFCFKEL; A*0301:AQMFFCFK; A*0301:DLAQMFFCFK; A*0301:LAQMFFCFK; as well as iTag-PE Tetramer (BD Coulter) to A*0201:AQMFFCFKEL; and A*24:DLAQMFFCF. Control represents results with a nonsense Dextramer (Immudex). Patient PBMC were washed in PBS and re-suspended at a concentration of 1 × 10^6^ cells/ml. 50 µl of sample was added to each well of a round bottom 96 well plate (Costar), and 5 µl of FcR Block reagent was incubated with cells at room temperature for 10 min. 10 µl of MHC Dextramer or iTag-PE Tetramer was added. The plate was vortexed and incubated for 20 min at room temperature in the dark. Following incubation, 10 µl anti-CD8-FITC (500 µg/ml clone T8; BD Coulter) was added, mixed and incubated for 30 min at room temperature. Cells were washed and re-suspended in PBS-fluorofix buffer (0.5 % paraformaldehyde). Data acquisition was obtained using a Fortessa flow cytometer running FACSDiva software. Data analysis was performed using FCS Express software. Results are based upon gating of CD8^+^ T cells and indicate the percent of cells positively labeled with specific tetramer.

#### T cell proliferation

PBMC were tested for peptide-specific proliferation as previously described [15]. PBMC were labeled with carboxyfluorescein succinimidyl ester (CFSE) and stimulated ex vivo with SVN53-67/M57 or control peptide (100 ng/ml over 48 h). Cells were stained for CD4 or CD8 and data acquired via FACS analysis and FCS Software as described above.

#### CD4^+^ and CD8^+^ separation

SureBeadsTM Protein G Magnetic Beads (Cat. 161-4023, Bio-Rad Laboratories, Inc.) were incubated with either LEAF CD8a (clone RPA-T8, Cat. 301018, BioLegened) or LEAF CD4 (clone OKT4, Cat. 317404, BioLegened). The antibody-bound magnetic beads were then incubated with 1 × 10^5^ PBMC. After bead-based CD4/CD8 separation, fluorochrome-labeled antibodies to CD8 (clone RPA-T8, FITC, Cat. 301050, BioLegend) and CD4 (clone OKT4, PE/Cy5, Cat. 317412, BioLegend) were added to remaining PBMC. The samples were acquired and analyzed by FACS as above. Analysis was based upon isolated gating of lymphocyte populations with specific CD markers as indicated. Sample purity is based upon the percent of specific cells removed from the starting population.

#### Cytokine mRNA isolation and quantification

Patient samples (1 × 10^5^ PBMC) were incubated with SVN53-67/M57 peptide at several concentrations (160–1600 ng/ml) in OpTmizer CTS complete serum-free media (Gibco) overnight at 37 °C in 96 well plates. After incubation, CD4^+^ and CD8^+^ T cells were separated from total PBMC by magnetic beads. Anti-human CD4 antibody (5 µg; clone OKT4) or anti-human CD8 antibody (5 µg; clone RPA-T8) (BioLegend, San Diego, CA) was added to Protein G Magnetic SureBeads (BioRad, San Diego, CA) and incubated for 60 min at room temperature with peptide-stimulated cells. Isolated cells were collected and washed in PBS. Total RNA was isolated from separated human CD4^+^ and CD8^+^ cells using the PureLink RNA Mini Kit (Life Technologies) according to manufacturer’s instructions. RNA was reverse transcribed using Superscript Vilo Master Mix (Life Technologies). cDNA concentrations were determined using a Nanodrop 2000 Spectrophotometer (Thermo Scientific). RT-qPCR was performed using a 7900HT Fast Real-Time PCR System (Life Technologies) with Power SYBR Green PCR Master Mix (Life Technologies). Primers for IFNγ had the sequences: 5′-GCATCCAAAAGAGTGTGGAG-3′ and 5′-ATGCTCTTCGACCTCGAAAC-3′. Glyceraldehyde-3-phosphate dehydrogenase (GAPDH) mRNA was used as an internal control, and GAPDH primers had the following sequences: 5′-GGTGAAGGTCGGAGTCAACGG-3′ and 5′-GAGGTCAATGAAGGGGTCATTG-3′. Reactions containing no cDNA were included in each run. Relative cytokine mRNA expression levels were calculated by the ΔΔ*C*_t_ method using RQ Manager software (Applied Biosystems). All samples were run in triplicate. The level of GAPDH gene expression served as an endogenous control. Data are represented as the ratio of the target gene/GAPDH normalized to unstimulated cells. Total mRNA values were expressed relative to PBMC RNA. An unpaired t test with Welch’s correction was applied to all of the data sets.

## Results

### Patient characteristics

Patient characteristics are listed in Table 1. All patients had previously undergone treatment, including: surgical resection, fractionated external beam radiation therapy and chemotherapy with temozolomide. Every patient had recurrent or progressive disease following failure of standard therapy at the time of entry. Five of nine patients were entered in the trial at first recurrence, and four patients were entered after two or more recurrences. Other previous treatments are listed in Table 1. Eight patients received the full complement of four priming doses of vaccine, and three patients received at least one booster dose in an extended dosing phase as well. One patient was not clinically evaluable due to rapid progression after the second dose of vaccine.

**Table 1:** Patient characteristics

Patient	HLA-A*		Age	Sex	Tumor^a^	Number of recurrences	Disease burden^b^	Prior treatments^c^	Priming doses	Booster doses
1	01	03	38	M	G1	1	+	S, R, T, S	4	+
2	01	02	45	F	G2	2	+++	S, R, T, S/C, B, SR	4	−
3	02	24	52	F	G2	1	+	S, R, T, S	4	–
4	01	02	34	M	G2	3	+++	S, R, T, SR, S, B, O	2^d^	–
5	02	24	58	M	G2	2	+	S, R, T, B, SR	4	+
6	02	02	48	F	G1	1	++	S, R, T, S	4	–
7	02	02	57	M	AG	2	+	S, R, T	4	+
8	01	02	61	M	G1	1	+	S, R, T, S	4	–
9	02	24	54	M	G2	1	+	S, T, S, R, T, S, SR	4	–

^a^G1, primary glioblastoma; G2, secondary glioblastoma; AG, anaplastic glioma

^b^Disease burden at first dosing: (–) no measureable contrast enhancement (C.E.); (+), measureable C.E., but <1 cm^3^; (++), >1 cm^3^ but ≤5 cm^3^ C.E.; (+++), >5 cm^3^ C.E

^c^ *S* surgery, *R* fractionated radiation therapy, *SR* stereotactic radiosurgery, *T* temozolomide, *B* bevacizumab, *O* other chemotherapy, *C* carmustine wafer implant

^d^N.E., not evaluable for clinical response due to tumor progression prior to third priming dose of vaccine

### Adverse events (AE) and toxicity

All nine patients were evaluable for the purposes of determining toxicity. SurVaxM demonstrated a good tolerability profile with no serious adverse events (SAE) during the prime-boost phase of up to four doses. The majority of AEs were grade one. Six of nine patients experienced at least one injection site reaction (all grade one) with localized erythema likely related to the vaccine components. Three patients reported fatigue (grades 1 or 2). Two patients experienced myalgias, probably related to the study regimen. Grade 1 lymphopenia was seen in three patients and leukopenia in three patients (grades 1 and 2). The only grade 3 AE, a seizure, was not related to the vaccine. During the extended dosing phase, one patient suffered acute renal failure (SAE) due to gabapentin toxicity with complete resolution following intravenous hydration. Renal biopsy showed no inflammatory tubular changes, immune complex deposition, glomerulonephritis or other evidence of immunologic toxicity.

### T cell responses

**Fig. 1:**
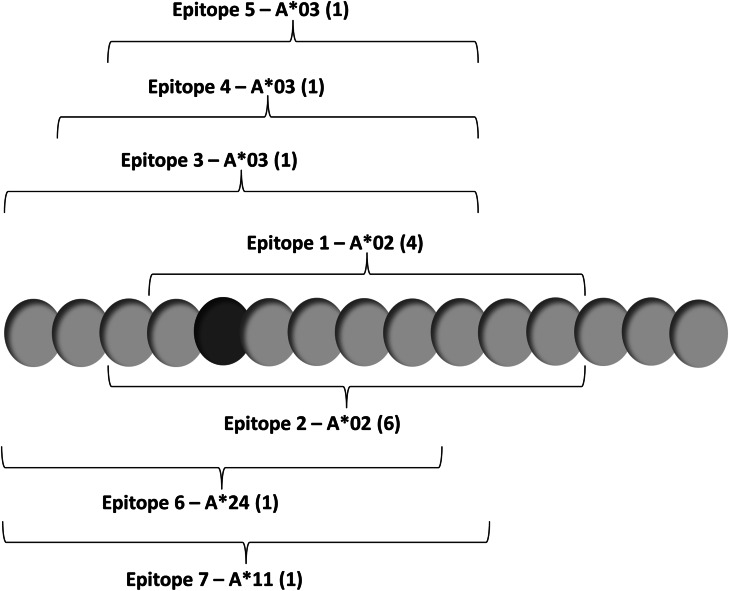
Long survivin peptide contained in SVN53-67/M57-KLH is shown with *brackets* indicating empirically confirmed immuno-reactive HLA-A*02, HLA-A*03, HLA-A*11 and HLA-A*24 epitopes within it. The position of the cysteine-to-methionine substitution (mimic) within the peptide is indicated (*dark gray*)

**Fig. 2:**
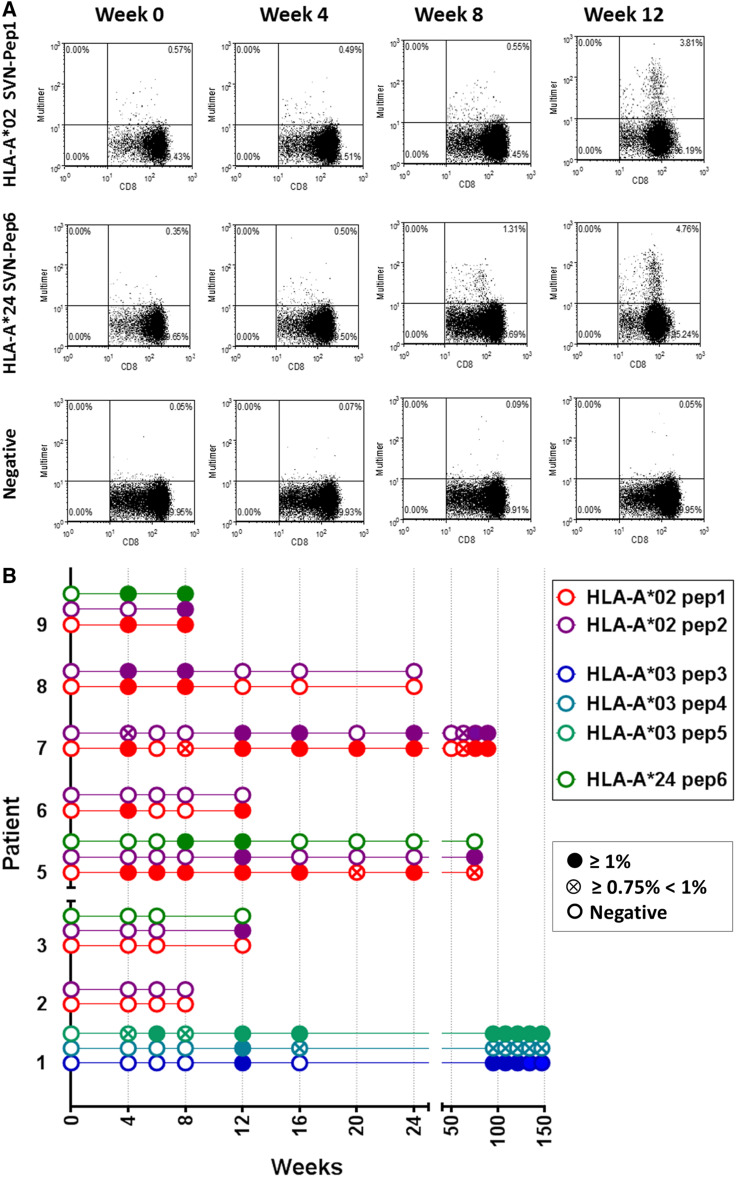
**a** FACS analysis of representative MHC class I dextramer assays of PBMC from a HLA-A*02/24 patient. Multimer binding of CD8^+^ T cells specific for survivin epitopes Pep1 and Pep6 (see Fig. 1) is shown. The *upper right quadrant* of each *panel* shows survivin multimer +/CD8^+^ gated T cells. Week 0 is pre-immunization blood sampling. Control represents results with a nonsense dextramer. **b** Binding of multimers to CD8^+^ T cell receptors in patients measured at weeks 8–150 following study entry. Data acquisition was performed by FACS analysis as revealed in **a**. Patient samples registering ≥1 % over baseline survivin-specific CD8^+^ T cells are shown as (*filled circle*). Patients not mounting an immune response to a particular peptide are shown as (*open circle*). Weakly positive patients registering ≥0.75 % and <1 % above baseline are (*circled times*). Custom-designed multimers included: Pep1, HLA-A*0201: QMFFCFKEL; Pep2, HLA-A*0201: AQMFFCFKEL; Pep3, HLA-A*0301: DLAQMFFCFK; Pep4, HLA-A*0301: LAQMFFCFK; Pep5, HLA-A*0301: AQMFFCFK; and Pep6; HLA-A*24: DLAQMFFCF. A negative control nonsense multimer was used to assess nonspecific binding. MHC Dextramer-PE (Immudex, Copenhagen, Denmark) to A*0201:QMFFCFKEL; A*0301:AQMFFCFK; A*0301:DLAQMFFCFK; A*0301:LAQMFFCFK; as well as iTag-PE Tetramer (BD Coulter) to A*0201:AQMFFCFKEL; and A*24:DLAQMFFCF

Individual epitopes contained within the long peptide are shown in Fig. 1. Survivin-specific T cell responses to vaccine were measured using synthetic MHC-peptide complexes (multimers) to CD8^+^ T cell receptors at baseline and after vaccination (Fig. 2a). Patient samples registering ≥1 % over baseline survivin-specific CD8^+^ T cells were recorded as positive (Fig. 2b). Values that registered ≥0.75 and <1 % above baseline were recorded as being weakly positive. Patients not mounting an immune response (<0.75 %) to at least one relevant peptide were recorded as being negative. Patients generally developed a measureable immune response beginning 8–12 weeks after initial vaccination. Two of three patients in the 3-month booster group experienced an increase in multimer-reactive CD8^+^ T cells over a period of 50–150 weeks, suggestive of established immune memory. In addition to HLA-A*02 and HLA-A*03 status, three patients had HLA-A*24 alleles permitting multimer analysis of HLA-A*24-restricted responses to vaccine. Two of three such patients had responses to the vaccine from both alleles.

### Humoral immune responses

**Fig. 3:**
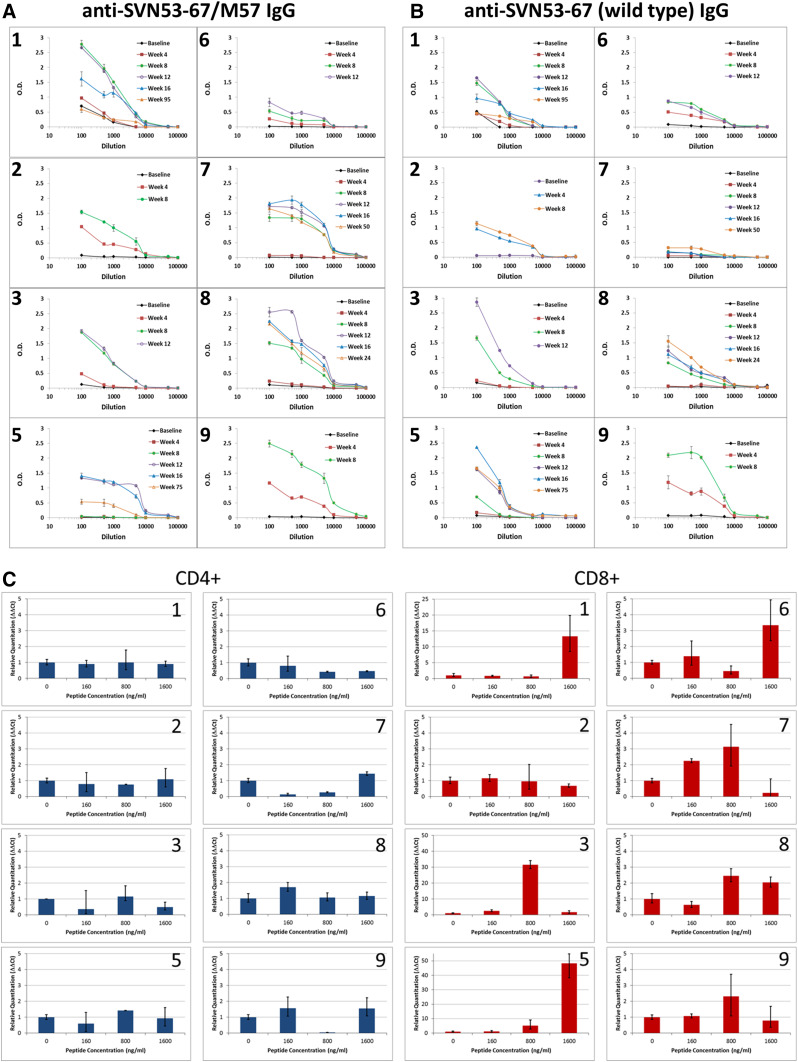
Antibodies produced in response to vaccination were measured as a potential biomarker. **a** IgG antibodies to SVN53-67/M57, and **b** IgG antibodies to SVN53-67 were measured in the serum of each patient by ELISA following prime-boost doses, and in those patients who entered an extended dosing phase. Seven patients produced significant levels (>1 O.D.) of SVN53-67/M57 (**a**) and six patients developed high titers (>1 O.D.) of SVN53-67 antibodies (**b**). One patient (#4) was not evaluable due to progression before all four prime-boost doses could be administered. **c** IFNγ mRNA levels in isolated CD4^+^ (*left*) and CD8^+^ (*right*) cells from individual vaccinated patients. Cells were stimulated in culture for 2 h with pooled overlapping survivin peptides (a.a. 53–67) at the indicated concentrations. qPCR of IFNγ mRNA was performed as described. Relative quantitation was performed using the ∆∆*C*_t_ method, and data were standardized to GAPDH mRNA expression and normalized to IFNγ mRNA levels in unstimulated cells. CD4^+^ and CD8^+^ cell populations were verified to be 94 and 98 % pure, respectively, by FACS analysis (data not shown)

IgG antibodies to epitopes within the survivin peptide (Fig. 3a) and to the corresponding wild-type survivin peptide (Fig. 3b) were detected in all evaluable patients with increasing titer over the course of the vaccination series. High titers (>1.0 O.D.) to the survivin vaccine peptide were detected in seven of eight patients. Six of eight patients developed high titers of antibodies that were cross-reactive to the corresponding wild-type survivin peptide. In addition, high titers (>1.0 O.D.) to KLH were detected in four patients and intermediate titers (>0.75 but ≤1.0 O.D.) were detected in two patients (data not shown).

### Cytokine production in CD4^+^ and CD8^+^ cells

CD4^+^ and CD8^+^ IFNγ mRNA levels were measured by qPCR using cells isolated by magnetic bead separation from PBMC obtained at week 12. Depletion of PBMC was confirmed to be 94 % (CD4^+^), and 98 % (CD8^+^) complete by FACS analysis (data not shown). Isolated cells were re-stimulated in culture with pooled overlapping survivin peptides. Induction of IFNγ mRNA was detected in CD8^+^ cells, but not in CD4^+^ cells (Fig. 3c). In general, IFNγ mRNA levels increased with higher peptide concentrations up to a maximum tested concentration of 1.6 µg/ml.

### T cell proliferation

**Fig. 4:**
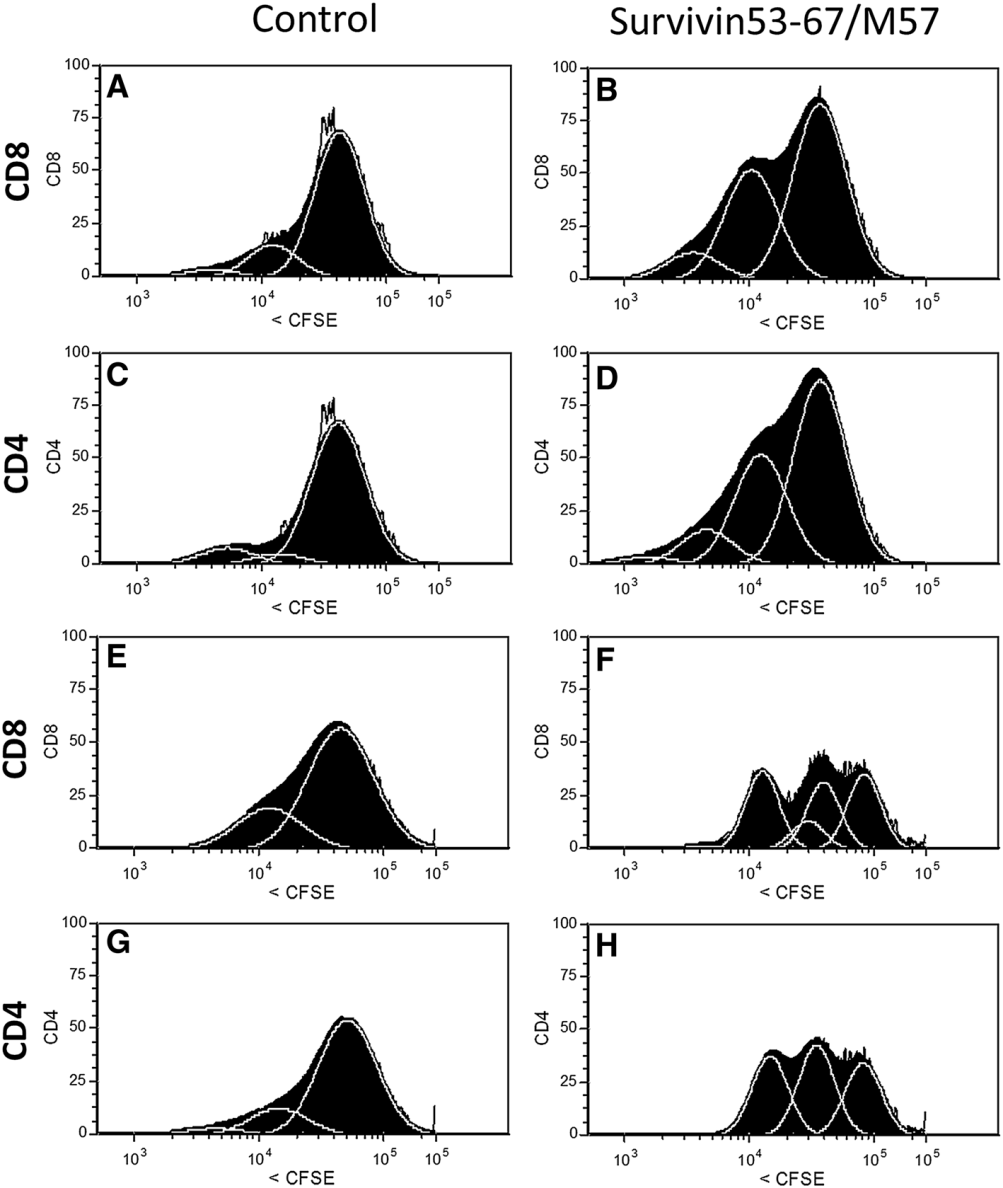
FACS analysis of CD8^+^ and CD4^+^ proliferation in CFSE dye-loaded PBMC obtained from two patients (patient #1; **a**–**d**) and (patient #7; **e**–**h**) 12 weeks following vaccination. Proliferation was measured in response to stimulation of cells using either the long (15 amino acid) survivin peptide mimic (SVN53-67/M57) or a 14 amino acid unrelated control peptide (seq: LEEKKQNYVVTDHC). Cell division is indicated by left-shifted peaks

Proliferation of CD4^+^ and CD8^+^ cells in response to ex vivo stimulation with the survivin peptide vaccine was measured by flow cytometry using CFSE-labeled PBMC (Fig. 4). Following co-culture with SVN53-67/M57, both CD4^+^ and CD8^+^ cells were stimulated to proliferate, whereas control peptide (irrelevant 14-mer) failed to stimulate proliferation.

### Tumor lymphocyte infiltrates

**Fig. 5:**
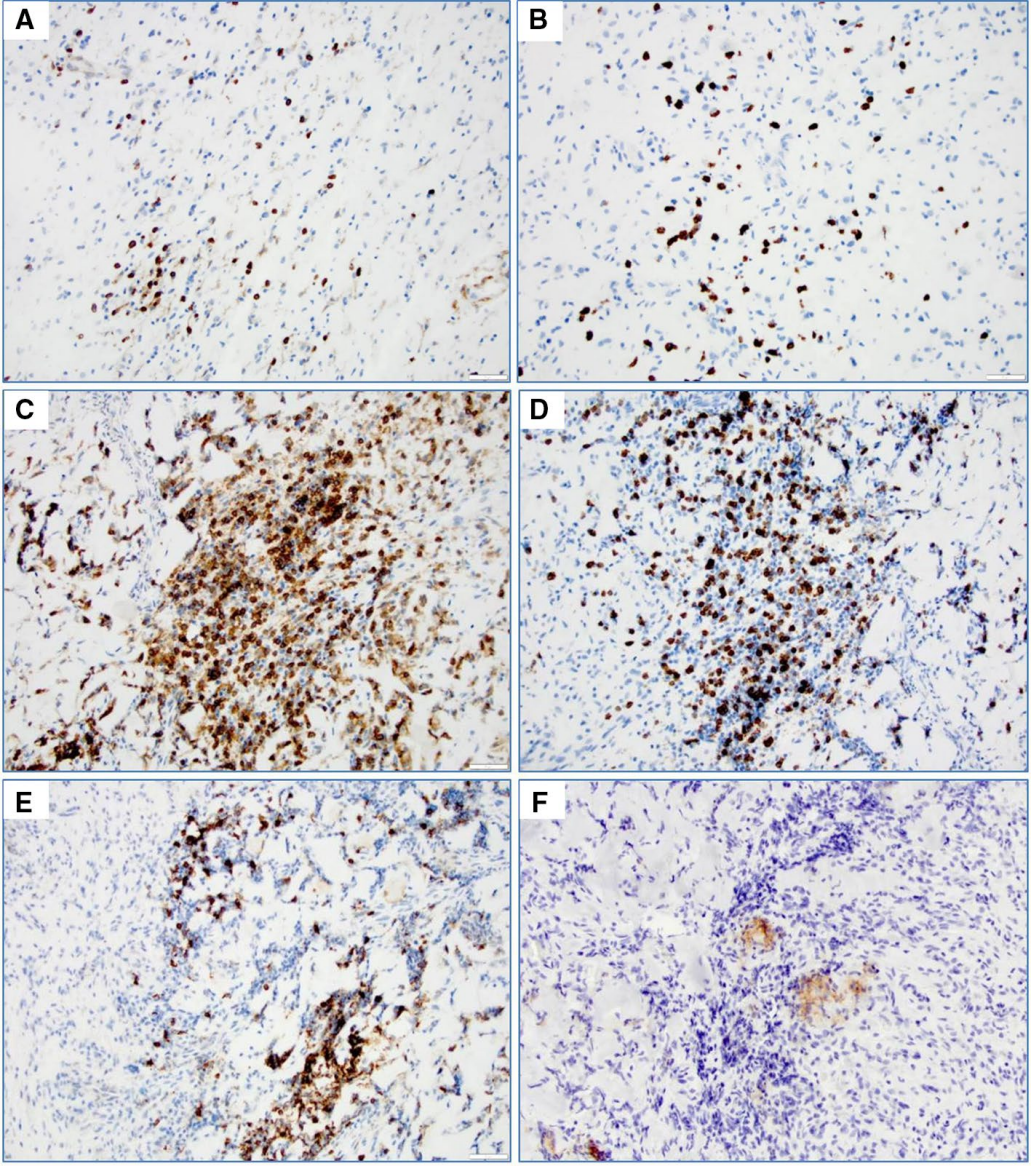
Immunohistochemistry of T and B cell markers (×200) in tissue sections of one patient (#8) with recurrent disease 5.6 months following protocol entry. **a** CD4^+^ and **b** CD8^+^ T cells are shown in representative fields of the patient’s glioblastoma prior to vaccine treatment. **c** CD4^+^, **d** CD8^+^, **e** CD20^+^ and **f** PD-L1^+^ cells within contiguous histologic sections of tumor after vaccine treatment and subsequent tumor recurrence

Three patients with tumor progression following the vaccination regimen underwent biopsy or surgical re-resection of their tumors with histologic evaluation. In all such cases, persistent survivin expression was observed within tumor cells (not shown). Tumors also displayed sparse focal islands of lymphocytes with co-localization of CD4^+^, CD8^+^ and CD20^+^ cells resembling lymph node-like germinal center organization (Fig. 5). In addition, small numbers of PD-L1^+^ cells were observed in these regions.

### Radiologic response, tumor progression and survival

**Fig. 6:**
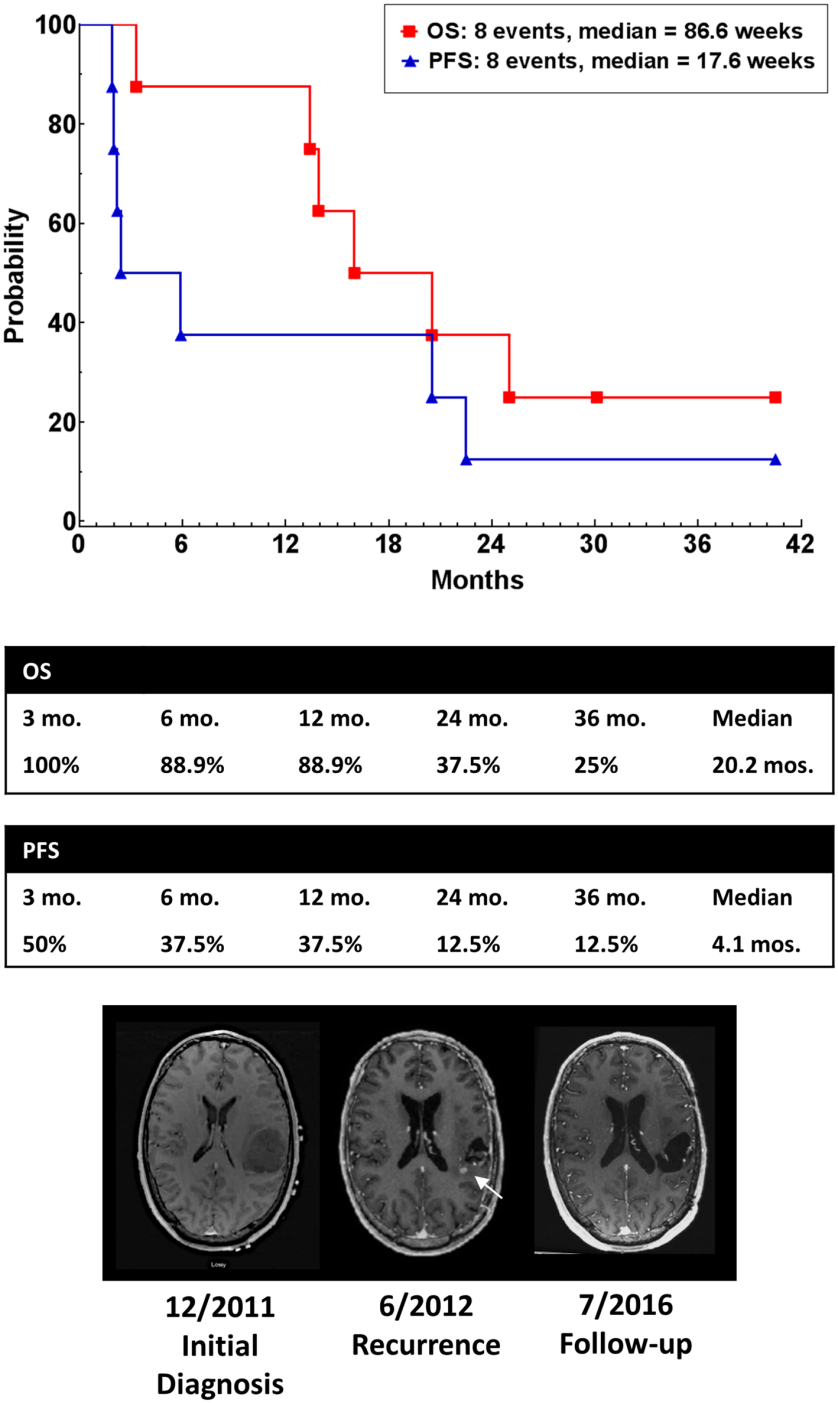
Progression-free survival (*filled triangle*) and overall survival (*filled square*) of patients. Median PFS was 17.6 weeks, and median OS was 86.6 weeks following study entry. MRI showing tumor at diagnosis (*left*), first recurrence (*center*) and 42-month follow-up (*right*)

One patient with recurrent glioblastoma had a CR and was alive and well with no evidence of disease 174 weeks following the first dose of vaccine. A second patient had a partial radiologic response, but subsequently progressed. Median PFS was 17.6 weeks, and median OS was 86.6 weeks in this study (Fig. 6). One patient (#5) died of unrelated medical causes with no tumor progression or mass effect (stable disease) documented on MRI scan obtained one week prior to death (PFS = OS = 88 weeks). Seven of nine patients survived more than 52 weeks following study entry.

## Discussion

The longest surviving patient in this study had a low but detectable titer of anti-survivin IgG in pre-immune serum. In line with this, previous studies have revealed circulating anti-survivin antibodies in patients with colon and lung cancer [9, 16] and in glioma patients as well. Similarly, T cell reactivity against survivin has been detected in cancer patients in the absence of specific vaccination [17–19]. Therefore, wild-type survivin is at least weakly immunogenic and re-stimulation of pre-existing immunity may be one aspect of the vaccine’s action.

Clearly, pre-existing anti-survivin immune responses do not completely eliminate tumor cells. We have previously demonstrated that immunologic tolerance to survivin antigens may be broken using molecular mimicry [11, 20]. SurVaxM contains a synthetic peptide with an amino acid substitution (SVN53-67/M57) that enhances HLA-A*0201 binding acting as a molecular mimic within this contextual background [11, 15, 21]. As a result, the core epitope induces a more potent immune response in humans than the corresponding wild-type survivin peptide [11]. The enhanced binding of SVN53-67/M57 to MHC-I molecules enables it to activate T cells that can cross-react with the wild-type tumor antigens leading to an anti-tumor response [11, 22]. Antigen modification may also aid directly in overcoming T cell tolerance since patients with non-HLA-A*02 haplotypes (i.e., HLA-A*03, HLA-A*11 and HLA-A*24) also developed CD8^+^ T cell responses to the vaccine in the current study.

The survivin peptide SVN53-67 contains multiple HLA-A*02 epitopes, as well as antigen-binding motifs for HLA-A*03; HLA-A*11, HLA-A*24, HLA-A*26, HLA-A*68, HLA-B*13, HLA-B*14, HLA-B*15, HLA-B*35, HLA-B*39 and HLA-B*44. We have confirmed in this clinical study that HLA-A*02, HLA-A*03 and HLA-A*24 patients generate survivin-specific humoral and cellular immune responses. In pre-clinical studies, we demonstrate that survivin peptide (SVN53-67/M57) stimulated CTL responses against autologous and allogeneic tumor cells from patients with HLA-A*2901, HLA-A*3002 haplotypes [11]. Moreover, in one patient treated under a compassionate use exemption survivin-specific HLA-A*11-restricted CD8^+^ T cells were generated in high numbers (data not shown). Consequently, the peptide vaccine is immunogenic in a relatively large segment of the population.

The survivin gene transcript is processed into a number of different mRNA splice variants. All of the various species noted to date retain exon 2, which encodes the region from which the peptide sequence in SurVaxM is derived. In addition to wild-type survivin, the survivin-2B and survivin–ΔEx3 transcripts, which are translated [23, 24], should also be targeted by immune responses generated by SurVaxM.

We used anti-survivin and anti-KLH IgG levels as a potential biomarker for assessing the immune response to vaccine. Eight patients were evaluable (≥2 priming doses) for immune response. Seven had high antibody titers to the survivin vaccine peptide, and 4 had high titers to KLH. Similarly, T cell responses to survivin were seen in seven patients following vaccination. Generally, patients with minimal disease burden had stronger antibody and T cell responses, although there were too few patients to make a definitive conclusion about the role of bulk disease in systemic immunosuppression. In addition, CD8^+^, CD4^+^ and B cell infiltrates were detected in recurrent tumor tissue obtained from patients following progression on-study. In one patient with a germinal center-like structure, PD-L1 was also expressed by adjacent glioma cells, suggesting that PD-1/PD-L1 interaction in the tumor microenvironment could be important for the immune effector response to SurVaxM in some patients [25].

Vaccination strategies utilizing individual CD8^+^ T cell epitopes alone do not routinely produce significant clinical responses. It is critical to stimulate CD4^+^ helper T cells simultaneously to potentiate the CD8^+^ T cell-driven anti-tumor response [11, 15, 20, 21, 26]. Specific CD4^+^ T cell stimulation results in the production of IFNγ, IL-2 and IL-4 which lead to more robust and sustained CTL activity [27, 28]. The presence of CD4^+^ support was detected by survivin peptide-induced CD4^+^ proliferation and can be inferred by the presence of IgM-to-IgG class switching. Generic peptides, such as tetanus, have been used to boost CD4^+^ T cell activity systemically. However, CD4^+^ T cells that are generated through exposure to tumor-associated antigens via antigen presentation on MHC class II are tumor-specific and play a more direct role in the tumor microenvironment [13, 14, 26, 27]. SVN53-67/M57-KLH activates multiple survivin-specific CD8^+^ T cell clones and stimulates survivin-specific CD4^+^ T cell proliferation [11, 12, 15, 21, 29]. Thus, in addition to stimulating CD8^+^ cellular and antibody-mediated immune responses, SurVaxM may provide important tumor-specific CD4^+^ helper support.

This first-in-human study demonstrated the safety, tolerability and immunogenicity of SurVaxM in patients with recurrent malignant glioma following failure of standard therapy. Progression-free survival was 17.6 weeks, and overall survival was 86.6 weeks. Comparatively, an analysis of eight consecutive phase II chemotherapy trials of patients with recurrent malignant glioma, historical PFS was 10 weeks and historical OS was 30 weeks [30]. Similarly, in a recent phase III clinical trial, which included an arm with physician’s choice chemotherapy in patients with recurrent glioblastoma, median PFS was 9 weeks and median OS was 25.7 weeks in the chemotherapy arm [31]. Currently, a larger multi-institutional, phase II clinical trial of SurVaxM plus standard therapy (Stupp protocol) is being conducted in patients with newly diagnosed glioblastoma.

## Abbreviations

AE: Adverse event
BIRC5: Survivin
CFSE: Carboxyfluorescein succinimidyl ester
CR: Complete response
CTCAE: Common terminology criteria for adverse events
CTL: Cytotoxic T lymphocytes
GAPDH: Glyceraldehyde-3-phosphate dehydrogenase
GM-CSF: Granulocyte macrophage-colony stimulating factor, sargramostim
HBSS: Hank’s balanced salt solution
IAP: Inhibitor of apoptosis protein
KLH: Keyhole limpet hemocyanin
KPS: Karnofsky performance status
OS: Overall survival
PFS: Progression free survival
PR: Partial response
RLT: Regimen-limiting toxicity
RPCI: Roswell Park Cancer Institute
SAE: Serious adverse event
SD: Stable disease
TCGA: The Cancer Genome Atlas
